# Clinical Outcome of Arthroscopic Reduction and Fixation by Pull Through Suture Technique in Tibial Spine Fractures

**DOI:** 10.7759/cureus.79902

**Published:** 2025-03-01

**Authors:** Ravi Shankar, Rohit Sherawat, Abhishek Sengupta, Nilesh Verma, Hitesh Garg, Harshvardhan Buddhist

**Affiliations:** 1 Orthopaedics and Traumatology, Vardhman Mahavir Medical College (VMMC) and Safdarjung Hospital, New Delhi, IND; 2 Orthopaedics, Vardhman Mahavir Medical College (VMMC) and Safdarjung Hospital, New Delhi, IND; 3 Orthopaedics and Trauma, Safdarjung Hospital, New Delhi, IND

**Keywords:** anterior cruciate ligament, arthroscopic, suture pull through technique, tibial spine avulsion, trauma

## Abstract

Background: Tibial spine fractures involving the avulsion of the anterior cruciate ligament (ACL) insertion compromise knee stability and often result in functional limitations if inadequately treated. These fractures are commonly categorized using the Meyers and McKeever classification, and treatment approaches have shifted from open reduction techniques to minimally invasive arthroscopic methods, such as the pull-through suture fixation technique. This study evaluates clinical outcomes of tibial spine fractures treated using arthroscopic pull-through suture fixation.

Methodology: A prospective study was conducted at a tertiary care hospital, including 20 patients (ages 15-55) with type III and IV tibial spine fractures. All patients underwent arthroscopic pull-through suture fixation. Preoperative and postoperative assessments included radiographs, Lachman, anterior drawer, pivot shift tests, and functional evaluations using Lysholm Knee and International Knee Documentation Committee (IKDC) scores. Follow-up assessments were performed at 6 weeks, 3, 6, and 12 months.

Results: The mean age of participants was 30.4 ± 9.8 years, with males constituting 75% of the cohort. Road traffic accidents (85%) were the predominant injury mechanism. The average time from injury to surgery was 10.25 ± 3.9 days, and union was achieved within 10.15 weeks on average. Functional outcomes showed significant improvement, with Lysholm scores increasing to 94.8 ± 1.7 and IKDC ratings achieving "normal" or "nearly normal" in 90% of patients at 12 months. Stability tests demonstrated marked improvements, with 95% of patients achieving Grade 0 on Lachman, anterior drawer, and pivot shift tests. Postoperative complications were minimal, with only one case of transient joint stiffness and one superficial wound infection.

Conclusion: Arthroscopic pull-through suture fixation effectively restores stability, function, and range of motion in type III and IV tibial spine fractures. The technique offers a reliable alternative to screw fixation, with fewer complications and a quicker recovery. These findings support its broader adoption as a preferred treatment method for displaced tibial spine fractures.

## Introduction

Tibial spine fractures, also known as avulsion fractures of the anterior cruciate ligament (ACL) insertion, involve the detachment of the tibial spine [1] from the proximal tibia with intact ACL insertion. The tibial spine serves as the primary attachment site for the ACL, which plays a crucial role in knee stability by preventing anterior translation of the tibia relative to the femur. Tibial spine fractures are most common in pediatric populations, with an estimated incidence of 3 per 100,000 children annually [2]. However, recent studies indicate that these fractures are also relatively common in adults [3], often linked to high-energy trauma such as road traffic accidents or sports injuries. When left untreated or inadequately managed, tibial spine fractures can result in significant disability, including knee instability, limited range of motion, and early onset of degenerative changes in the joint.

First described by Poncet in 1875, tibial spine fractures are relatively rare injuries, accounting for 2-5% of all pediatric knee injuries [4]. The injury mechanisms vary between pediatric and adult populations. In children, tibial spine fractures tend to occur from low-energy impacts, such as falls from a bicycle, due to the weaker resistance of the growth plate [4,5]. In contrast, adults often experience these injuries from high-energy trauma, including road traffic accidents and competitive sports, resulting in a greater incidence of associated soft tissue injuries, such as meniscal tears or collateral ligament sprains [6]. The varying mechanisms and presentation across age groups underscore the complexity of this injury and the need for tailored treatment approaches.

Tibial spine fractures are commonly classified using the Meyers and McKeever system [7], which categorizes fractures based on their displacement and associated rotation. The classification was later modified by Zaricznyj [8] to include a fourth type, which involves the comminution of the fracture fragment. Types III and IV fractures typically require surgical intervention due to the high risk of nonunion or malunion, which can lead to chronic instability and functional impairment [9-11].

Historically, tibial spine fractures were managed with open reduction and internal fixation. While effective in stabilizing the fragment, open reduction methods are associated with higher complication rates, such as postoperative stiffness, extended recovery time, and the need for additional surgery to remove hardware. The advent of arthroscopic techniques revolutionized the management of tibial spine fractures, offering a minimally invasive alternative that allows for direct visualization of the fracture site [12-17].

The primary goal of this study is to evaluate the clinical outcomes of tibial spine fractures treated with the arthroscopic suture pull-through fixation technique. Functional outcome was assessed using the International Knee Documentation Committee (IKDC) and Lysholm score [18,19].

## Materials and methods

This prospective study was conducted over two years at the Department of Orthopedic Surgery at CCS Hospital, Swami Vivekanand Subharti University. Twenty patients who presented with type III and IV tibial spine fractures underwent arthroscopic fixation using the pull-through suture technique.

Participants included male and female patients aged 15 to 55 years. All patients had sustained tibial spine fractures with anterior knee instability and were eligible if they presented within four weeks of injury. Exclusion criteria included those with significant degenerative changes, associated injuries to the posterior cruciate ligament, or other significant ipsilateral femoral or tibial fractures. Ethical approval was obtained, and all patients provided written informed consent. Their demographic data was meticulously documented to ensure accurate case tracking.

A comprehensive history was recorded for each patient, emphasizing the duration and mechanism of injury (e.g., falls, sports-related incidents, vehicular accidents) and any prior treatments received. Two senior orthopedic consultants conducted detailed knee examinations to confirm the diagnosis, focusing on ACL integrity and tibial stability assessments. The Lachman test, anterior drawer test, and pivot shift test were performed to assess knee stability. Knee range of motion (ROM) was measured with a goniometer, while the function was assessed using the Lysholm Knee Score and IKDC Score.

Anteroposterior and lateral X-rays confirmed bone fragmentation at the ACL's tibial attachment, and an MRI was used to detect additional intra-articular or soft-tissue injuries. The diagnosis of ACL avulsion fractures was based on anterior instability in physical exams and radiographic evidence.

All patients were operated on by two senior orthopedic surgeons. Following induction of general, spinal, or epidural anesthesia, the patient was positioned supine on the operating table. A well-padded tourniquet was placed high on the thigh to control blood flow. The thigh was secured in a holder, and the table was adjusted to allow knee flexion between 110°-120° for optimal access to the tibial tunnels during drilling. The surgeon stood adjacent to the patient, with the arthroscopy tower positioned directly in front.

Diagnostic arthroscopy was performed via a standard anterolateral portal. Continuous irrigation was used to clear the joint and fracture site of any hematoma. A standard anteromedial (AM) portal was created, and a suture cannula was introduced to allow further instrumentation. Any chondral or meniscal injuries identified were addressed according to standard treatment protocols. The tibial spine avulsion fracture was visualized, and the fracture type was confirmed with probing. An accessory trans-patellar tendon portal was established for additional access.

The avulsed fragment was reduced using an ACL tibial guide. An 18-gauge intravenous (IV) cannula loaded with no. 1-0 PDS suture was introduced into the joint at an angle parallel to the medial tibial condyle, puncturing the ACL at its attachment to the bony fragment slightly posteriorly. Once the needle tip exited the lateral ACL, the PDS suture was grasped and retrieved through the AM portal. The IV cannula was withdrawn, and the PDS limb on the medial ACL side was similarly retrieved through the AM portal. The PDS suture was replaced with no. 1 fiber wire by suture shuttling, and both ends were clamped with artery forceps.

The ACL tibial guide was then positioned via the trans-patellar portal at the lateral edge of the avulsion site. A small incision (1 cm) was made near the entry of the guide sleeve, just medial to the tibial tuberosity. A 1.8-mm K-wire was drilled to create a tibial tunnel, with the tip visualized at the fracture crater. The K-wire and guide were then removed. An 18-gauge IV cannula preloaded with PDS was passed through the tunnel. The PDS was advanced and retrieved through the AM portal using a suture grasper, allowing the fiber wire exiting from the lateral ACL to be shuttled through the tibial tunnel. A second tunnel was drilled at the medial edge of the crater, preserving a 1-cm bone bridge between the tunnels. The other fiber wire end exiting from the medial ACL border was similarly shuttled through this second tunnel.

Finally, the fiber wire ends were secured over the bony bridge or a suture disc, with the knee positioned in 30° flexion to verify adequate reduction. The knee was then fully extended to confirm no impingement of the intercondylar roof with the fixed tibial spine fragment. Incisions were closed in layers to complete the procedure.

Post-surgery, the knee was stabilized with a compression dressing and brace. After three days, patients began walking with a walker and doing quadriceps exercises. Knee mobilization started after two weeks, aiming for 90° of flexion within three weeks. Antibiotics were given for 48 hours, with NSAIDs and opiates as needed.

The knee was immobilized in an extension brace for two weeks, and non-weight-bearing ambulation started on day two with crutches or a walker. Closed-chain exercises began by week three, with partial weight-bearing allowed after four weeks and full weight-bearing once healing was confirmed. Isometric quadriceps exercises were done to prevent muscle atrophy. Sports were resumed after six months when stability, ROM, muscle strength, and proprioception were restored.

Patients were evaluated clinically and radiographically at 6 weeks, 3, 6, and 12 months post-operatively. X-rays (AP and lateral views) were taken at six weeks and three months to monitor fracture healing, with union confirmed if no fracture line was visible. At six months, an independent investigator assessed anteroposterior laxity using the Lachman and anterior drawer tests, and knee range of motion was measured with a goniometer. Knee function was evaluated using the Lysholm and IKDC scores preoperatively and at 6 months and 12 months.

## Results

This study included a total of 20 patients with tibial spine fractures who were treated with arthroscopic reduction and fixation using the pull-through suture technique. Patients ranged in age from 15 to 55 years, with a mean age of 30.4 ± 9.8 years. The majority of participants were male (75%), with 15 male and 5 female patients, reflecting a higher incidence of this type of injury in males. The predominant mechanism of injury was road traffic accidents, which accounted for 85% of cases (n=17), followed by sports injuries, comprising 15% of cases (n=3) (Table 1).

**Table 1:** Demographic distribution of the patients.

Category	Subcategory	Frequency	Percentage (%)
Age Group	15-25 years	5	25
	26-35 years	10	50
	>36 years	5	25
Gender	Male	15	75
	Female	5	25
Side Involved	Right	12	60
	Left	8	40
Mode of Injury	Road Traffic Accident	17	85%
	Sports Injury	3	15%
Duration Between Injury and Surgery	0–5 Days	2	10%
	6–10 Days	9	45%
	11–15 Days	7	35%
	More than 15 Days	2	10%
Fracture Classification (Meyers & McKeever)	Type III	16	80%
	Type IV	4	20%

The fractures were categorized based on the Meyers and McKeever classification system, with 80% (n=16) identified as Type III and 20% (n=4) as Type IV. Physeal status indicated an open physis in 4 patients and a closed physis in 16 patients (Table 2). In this study, the time from injury to surgery averaged 10.25±3.9 days, with 10% of cases at 0-5 days, 45% at 6-10 days, 35% at 11-15 days, and 10% over 15 days. The average union time in our study was 10.15 weeks, with a shorter union time in the open physis group (9.6 weeks) compared to the closed physis group (10.7 weeks).

**Table 2:** Physis type and radiological union time.

Category	Sub-category	Frequency	Percentage	Union Time (weeks)
Physis Type	Open	4	20%	9.6
	Closed	16	80%	10.7
	Total	20	100%	10.15 (mean)

Preoperatively, all patients demonstrated poor functional scores as measured by the Lysholm Knee Score and the IKDC score, which were below 65 and classified as abnormal or severely abnormal, respectively. By the final 12-month follow-up, there was a statistically significant improvement in these scores. The mean Lysholm Knee Score increased from a poor preoperative level to 94.8 ± 1.7, with 95% of patients reaching the "excellent" category (score of 95-100). Similarly, the IKDC score improved from preoperative ratings of "abnormal" or "severely abnormal" in all patients to "Normal" (A) in 90% of patients and "Nearly Normal" (B) in 10% at 12 months (Table 3).

**Table 3:** Preoperative and final follow-up scores for Lysholm knee and IKDC rating. IKDC: International Knee Documentation Committee

Category	Score	Preoperative	Final Follow-Up
Lysholm Knee Score	Excellent (95-100)	0 (0%)	19 (95%)
	Good (84-94)	0 (0%)	1 (5%)
	Fair (65-83)	0 (0%)	0 (0%)
	Poor (<65)	20 (100%)	0 (0%)
IKDC Rating	Normal (A)	0 (0%)	18 (90%)
	Nearly Normal (B)	0 (0%)	2 (10%)
	Abnormal (C)	4 (20%)	0 (0%)
	Severely Abnormal (D)	16 (80%)	0 (0%)

In terms of knee stability, functional testing using the anterior drawer, Lachman, and pivot shift tests showed substantial improvements. Preoperatively, patients exhibited increased anterior tibial translation on anterior drawer, Lachman, and pivot shift testing. At the one-year follow-up, 19 patients (95%) displayed no instability on these tests, achieving a Grade 0 on all the tests with only one patient having a Grade I anterior drawer, Lachman, and pivot shift test, indicating mild residual laxity.

The postoperative range of motion (ROM) was another key measure of functional recovery. At three months, 70% of patients had ROM limitations, with knee flexion below 130 degrees. However, ROM progressively improved over subsequent follow-ups. At 12 months, 90% of patients had achieved flexion within 130 to 150 degrees, and all patients could fully extend their knees, indicating successful restoration of functional movement.

Complications were minimal in this cohort. Only two cases of postoperative complications were reported: one patient experienced a superficial wound infection, which resolved with antibiotic treatment, and another developed transient joint stiffness, which improved with physiotherapy. Importantly, no cases of nonunion, ACL deficiency, or instability were reported at the final follow-up.

## Discussion

The present study included 20 patients and a 12-month follow-up period. It was conducted at a tertiary care teaching hospital in which patients with tibial spine fractures (McKeever type III and type IV) treated by arthroscopic reduction and fixation by pull-through suture technique without associated injuries were evaluated. Post-operatively, the patients were followed at 3, 6, 12 months, with a mean follow-up of 10 ± 2.8 months.

In the present study, the average age of the patients was 30.4 years, closely aligning with the mean age reported in other studies, such as Sapre et al. [20] with a mean age of 29.2 years and Yuan et al. [21] with a mean age of 34.2 years. This suggests that the patient demographics in our study are comparable to those in global studies on tibial spine fractures. The mean age of male patients was 29.6±11.2 years, while female patients had a mean age of 32.6±3.3 years, which reflects the gender imbalance in our cohort, as only five female patients were included, in contrast to other studies where females outnumber males, such as in the study by Yuan et al. [21]

We used an indigenously developed knee laxity measurement device, the "Laxometer," to assess objectively anteroposterior knee laxity before and after surgery. Based on the principles of the commercially available Rolimeter, this device proved effective in measuring anterior translation in ACL-deficient knees, similar to the KT-8000 meter as reported by Singhal et al. [22] in his study. At the final follow-up, the Laxometer data showed side-to-side differences of less than 3 mm in all patients, indicating improved anteroposterior stability compared to preoperative values.

The study showed no significant difference in the functional outcomes between age groups, which is consistent with previous research. Pandey et al. [23] reported that both open and closed physis groups had favorable outcomes after arthroscopic fixation, with Lysholm and IKDC scores significantly improving. Similarly, our study found no significant difference in Lysholm scores 95±1.4 vs. 94.75±1.8 or IKDC scores 95.2±1.2 vs. 93.7±1.5 between patients with open or closed physis, respectively, and range of motion was also similar, i.e., 139.2±0.95 vs 137.3±2.4 degrees between these groups.

Regarding injury and surgery intervals, the average duration from injury to surgery in our study was 10.25 days, which aligns with the injury-surgery intervals as reported by Pandey et al. [23] and Wang et al. [24].

The average operating time in our study was 70 minutes, slightly shorter than the 80 minutes reported by Wang et al. [24], with the variation influenced by whether a consultant or a trainee doctor performed the surgery. Radiographically, the average union time in our study was 10.15 weeks, ranging from 9 to 12 weeks. Yuan et al. [21] and Liao et al. [25] also saw similar findings, with an average union time of 12 weeks.

At the final follow-up, there were no signs of instability or ACL deficiency, and all patients had negative Lachman and pivot shift tests. The most common mode of injury was road traffic accidents (85%), followed by sports injuries (15%), and the functional outcomes for both groups were similar. The study also found no significant difference in Lysholm and IKDC scores based on fracture type (Type III vs. Type IV) or gender.

Post-operatively, the IKDC rating improved significantly, with 90% of patients achieving a "normal" or "nearly normal" rating at the final follow-up. In terms of complications, there were no major postoperative issues such as infection, deep vein thrombosis, or neurovascular deficits. One patient experienced arthrofibrosis, which improved after physiotherapy, which contrasts with the results shown by Montgomery et al. [26], who reported nine cases of severe restriction of knee range of motion, and another had a superficial wound infection, which was resolved with antibiotics.

## Conclusions

Arthroscopic reduction and fixation of tibial spine fractures using the pull-through suture technique yield favorable outcomes regarding knee stability, function, and range of motion. The low complication rate and lack of residual ACL deficiency in most cases further support this technique as an effective treatment option for displaced tibial spine fractures in both young and adult patients. Continued advancements in suture-based arthroscopic techniques hold promise for improved outcomes in managing these complex injuries.
